# The Effect of Chronic Bacterial Prostatitis on Semen Quality in Adult Men: A Meta-Analysis of Case-control Studies

**DOI:** 10.1038/srep07233

**Published:** 2014-11-28

**Authors:** Yonggang Shang, Chengcheng Liu, Dong Cui, Guangwei Han, Shanhong Yi

**Affiliations:** 1 Department of Urology, Xinqiao Hospital, Third Military Medical University, Chongqing, 400037, China

## Abstract

Chronic bacterial prostatitis (CBP) is caused by bacterial infection and maintains a condition of lower urinary tract infection. It may be a cause of male infertility. However, studies showed inconsistent results regarding the effect of CBP on several parameters of semen. Hence, we conducted a meta-analysis to examine the effect of CBP on basic semen parameters. A systematic review was conducted with Medline, PubMed, EMBASE, and two Chinese databases (CNKI and WANG FANG) to identify relevant studies that involved the effect of CBP on semen parameters up to July 2014. Both RevMan5.2 and STATA 12.0 software were used for the statistical analysis. Based on the inclusion and exclusion criteria, seven studies were included. The study illustrated that sperm vitality, sperm total motility, and the percentage of progressively motile sperm from CBP patients were significantly lower than controls (SMD(95%CI) −0.81[−1.14, −0.47], −1.00[−1.28, −0.73], −0.41 [−0.70, −0.12], P<0.05, respectively). However, CBP had no significant effect on semen volume, sperm concentration and the duration of semen liquefaction. In summary, our study revealed that there was a significant negative effect of CBP on sperm vitality, sperm total motility, and the percentage of progressively motile sperm. Additional, studies with larger number of subjects are needed.

Chronic prostatitis is a common disease in men, and the incidence is gradually increasing^1^. In 1999, the National Institutes of Health (NIH) classified prostatitis into the following four categories: I-- acute bacterial prostatitis; II-- chronic bacterial prostatitis; III-- chronic prostatitis/chronic pelvic pain syndrome (CP/CPPS); and IV-- asymptomatic inflammatory prostatitis^2^.

Among these four types,chronic bacterial prostatitis (CBP) accounts for approximately 5 to 10% of all the symptomatic cases^3^, and about 30% of adult men suffer from chronic bacterial prostatitis that present with recurrent urinary tract infections^4^. CBP is caused by bacterial infection and maintains a condition of lower urinary tract infection that triggers multiple disorders^5^. Although a bacterial infection is the primary cause, the optimum treatment is still unknown. Antibiotics and other drugs cannot fully enter the prostate tissues, which limits the efficacy of treatment^6^. Because of the complicated patient states, ineffective treatments and high relapse rate, CBP seriously influences the health of adult males^7^.

In 1995, an investigation showed that male infertility affected approximately 15% of the couples in Europe^8^. Poor semen quality may be the most common cause of male infertility, and there is a general consensus that reduced fertility and poorer semen quality may be the result of male accessory gland infection^9^. Studies have shown that the pathogenic bacteria, leukocytes, cytokines and reactive oxygen species (ROS) might be the primary mechanisms of infertility resulting from male accessory gland infection^10^,^11^, and broad-spectrum treatment could reduce the density of leukocytes in semen and improve the ejaculates quality^12^.

Many studies have shown the negative effect of CBP on basic semen parameters by decreasing sperm total motility, the percentage of progressively motile sperm and delaying the duration of semen liquefaction^13^,^14^. However, others studies showed contradictory results^15^,^16^. Hence, the aim of our study was to assess the effect of CBP on basic semen parameters by conducting a meta-analysis of relevant case-control studies.

## Methods

### Literature Search Strategy

An electronic literature search was conducted with Medline, PubMed, EMBASE, and two Chinese databases (CNKI and WANG FANG) to identify relevant case-control studies published up to July 2014. The following keywords or phrases were used: “prostatitis”, “chronic prostatitis”, “chronic bacterial prostatitis”, “II prostatitis”, in combination with “male infertility”, “semen”, "sperm”, “spermatozoa”, “semen analysis”, “spermatozoon”, “semen parameters”, “sperm quality” with no restrictions. In addition, the reference lists of the retrieved articles were reviewed, and we contacted the authors of the primary studies for additional information.

### Inclusion Criteria

The following inclusion criteria were used: 1. studies with a case-control study design; 2. patients meeting the diagnostic criteria for CBP according to the NIH classification or the traditional definition of CP; 3. the controls should be healthy adult males; 4. semen samples were obtained before therapeutic intervention; 5. available data that could be extracted from the article or obtained by calculation.

### Exclusion Criteria

If the studies met the following criteria, they would be excluded: 1. review articles, case reports, animal researches and no case-control design; 2. studies regarding other types of CP and patients who were previously diagnosed with other diseases that could lead to infertility; 3. patients with a mean age of less than 12 years or more than 60 years.

### Study Selection

Initially, we reviewed the titles and abstracts to ascertain the potential fit with the inclusion criteria. In the presence of uncertainty regarding the relevancy, a subsequent full-text assessment was conducted. Because the data included in this study were retrieved from the literature, ethical approval from ethics committees was not needed.

### Data Extraction and Validity Assessment

The following information was extracted: last name of the first author, publication year, mean age of the participants, study population, number of cases and controls, and basic semen parameters. The literature retrieval was conducted in duplicate by two independent authors.The controversial issues were resolved by discussion.

Two authors completed the quality assessment based on the primary criteria for non-randomized and observational studies of the Newcastle- Ottawa Quality Assessment scale (NOS) for meta-analyses^17^.

### Statistical Analysis

The analysis was performed using RevMan5.2 and STATA 12.0 software. The standard mean differences (SMDs) and the corresponding 95% confidence intervals (CIs) were used to measure the effect of CBP on the basic semen parameters. Homogeneity test was performed with the use of Q statistic and the I^2^ statistic. A random-effects model or, in the absence of heterogeneity, a fixed-effects model was used to combine the study-specific SMDs and compute the summary SMD. Additionally, we conducted a sensitivity analysis to investigate the influence of a single study on the overall risk estimate by omitting one study in turn. In addition, publication bias was detected by Begg's and Egger's test. In our study, if the P value was less than 0.05, it was considered as statistically significant.

## Results

### Characteristics of the Included Studies

**Figure 1:**
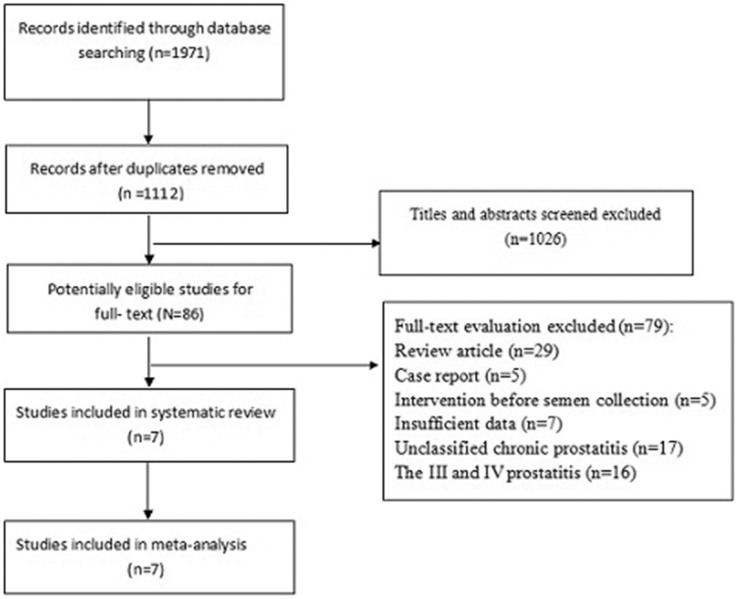
Flow chart of the study selection.

Figure 1 presents a flow chart showing the study selection process. We initially identified 1971 potential studies from the above databases, andmost of them were excluded because they were not case-control studies or because the exposure or endpoint was not relevant to our analysis. 7 eligible studies^3^,^13^,^14^,^15^,^16^,^18^,^19^, involving 249 cases and 153 controls, were included. Table 1 summarizes the general data from the included 7 studies.

### Meta-Analysis

**Figure 2:**
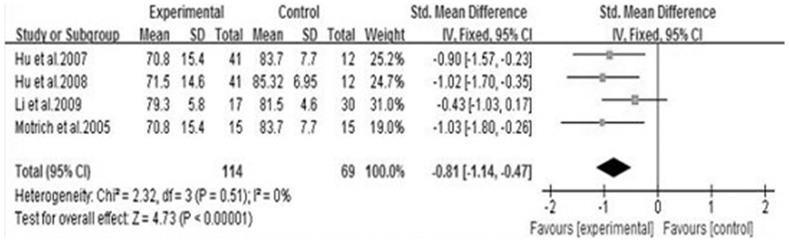
Forest plot of the effect of CBP on sperm vitality.

**Figure 3:**
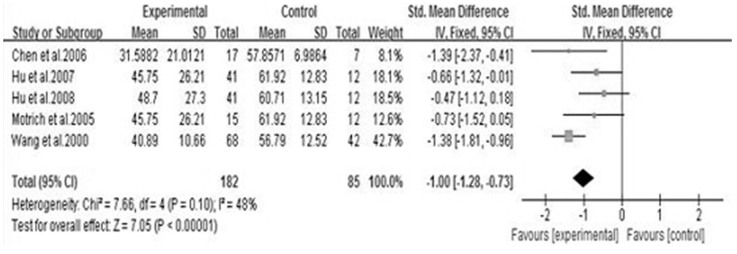
Forest plot of the effect of CBP on sperm total motility.

**Figure 4:**
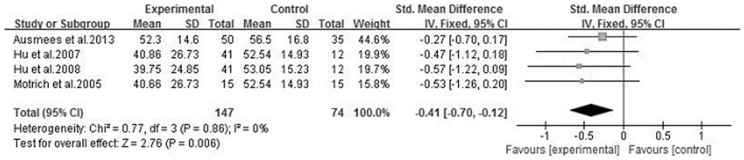
Forest plot of the effect of CBP on the percentage of progressively motile sperm.

**Figure 5:**
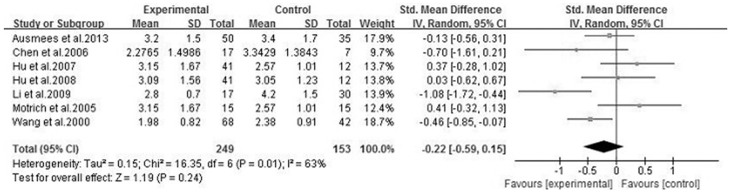
Forest plot of the effect of CBP on semen volume.

**Figure 6:**
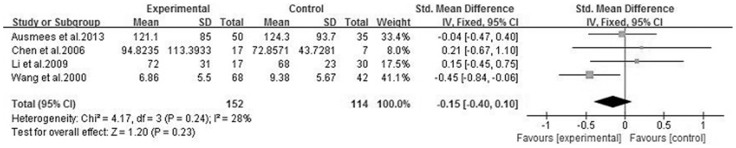
Forest plot of the effect of CBP on sperm concentration.

**Figure 7:**
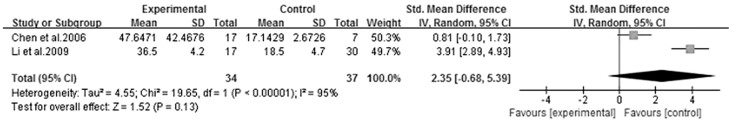
Forest plot of the effect of CBP on the duration of liquefaction.

We analyzed 6 semen parameters, and the results suggested that sperm vitality, sperm total motility and the percentage of progressively motile sperm from the patients with CBP were significantly lower than controls, and the pooled SMDs (95%CIs) were -0.81 [−1.14, −0.47], −1.00 [−1.28, −0.73], −0.41 [−0.70, −0.12], P<0.05, respectively. There was no evidence of significant heterogeneity among these studies (P>0.05, I^2^<50%) (Figures 2, 3 and 4). However, semen volume, sperm concentration and the duration of semen liquefaction in CBP patients were not significantly different from controls (SMDs (95%CIs) were −0.22 [−0.59,0.15], −0.15 [−0.40, 0.10], 2.35 [−0.68,5.39], P>0.05, respectively). There was evidence of significant heterogeneity among these studies (P<0.05, I^2^>50%) except regarding the sperm concentration analysis (P>0.05, I^2^<50%) (Figures 5, 6 and 7).

### Sensitivity Analysis

**Figure 8:**
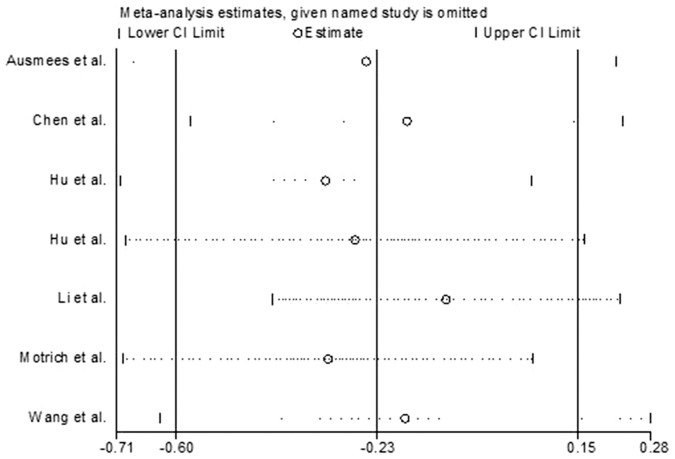
The plot of result of sensitivity analysis.

We omitted one study sequentially, and the calculated combined SMD for the remaining studies yielded consistent results. In the overall meta-analysis, no single study significantly changed the combined results, which indicated that the results were statistically stable and reliable (Figure 8).

### Publication Bias

**Figure 9:**
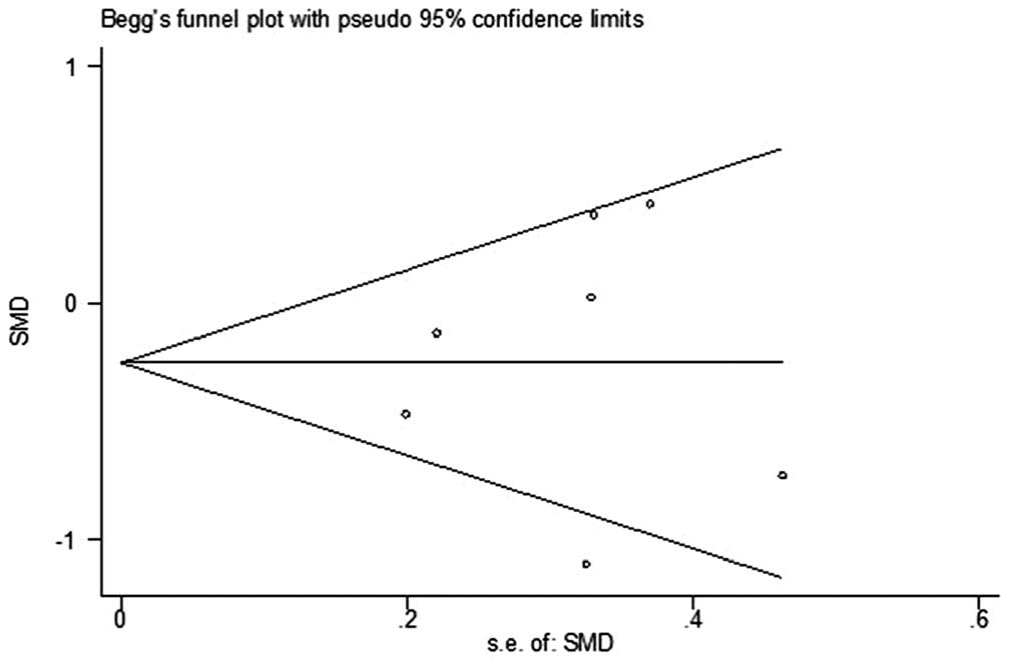
Begg's funnel plot of the included studies for publication bias.

Begg's funnel plot did not show any substantial asymmetry (Figure 9). Egger's regression test indicated little evidence of publication bias (P = 0.780).

## Discussion

Our meta-analysis of 7 case-control studies revealed a significant negative effect of CBP on sperm vitality, sperm total motility, and the percentage of progressively motile sperm. However, the relationships between CBP and semen volume, sperm concentration and the duration of semen liquefaction were not identified. The evidence of heterogeneity was observed across the studies, which was partially explained by the following facts: 1. the inconsistent methods of semen parameters measurement; 2. the inconsistent standard units for measuring semen parameters in different articles; 3. different populations existed in the studies; 4. all the studies used a case-control study design, and most of studies were in Chinese.

According to the presence or absence of white blood cells in the expressed prostatic secretion (EPS), the presence or absence of bacteria in the EPS, and the patient's clinical symptoms, prostatitis was classified into four categories^20^. Chronic bacterial prostatitis, category II, is a common cause for recurrent urinary tract infections in adult men, and Gram-negative bacilli (particularly E.coli) was responsible for the great majority of cases^21^. Bacterial infection can release toxins and inflammatory factors into the prostate and cause an autoimmune reaction that can lead to the deterioration of semen quality and sperm damage^22^. Additionally, the up- regulation of the apoptotic protein, Omi/HtrA2, in sperm causes poor semen quality^19^.

In the clinic, sperm vitality is used to make a distinction between necrozoospermia and total asthenozoospermia^23^, and to evaluate the cellular membrane integrity^24^. According to the WHO criteria, when the percentage of immotile spermatozoa is more than 40%, it is clinically important to verify the proportion of live spermatozoa^25^. Reactive oxygen species (ROS) and inflammatory cytokines can reduce the sperm vitality. In the prostate, inflammatory reaction may increase the oxidative stress, which leads to impairment of sperm cells^26^. In our meta-analysis, the results of four studies were inconsistent^3^,^14^,^15^,^19^, one reported that CBP did not have a negative effect on sperm vitality^15^, whereas the result of our study showed that sperm vitality from the patients with CBP was significantly lower than controls (Figure 2).This finding was consistent with the result of the previous study^26^.

Sperm motility is essential for fertilization, and there is a moderate correlation between sperm motility and sperm chromatin as well as mitochondria, structure and function^27^,^28^. Sperm motility is strongly dependent on the mitochondrial oxidative phosphorylation system^29^. Thus, changes in DNA and/or mtDNA genes can lead to decrease sperm motility. Progressive motility is a vital functional characteristic of sperm, and it measures the ability of sperm to penetrate into, and migrate through, both cervical mucus and oolemma^30^. In 2010, WHO stipulated the limit for total motility at 40% and progressive motility at 32%, and males with sperm motility values below these threshold values were classified as asthenozoospermic^25^.

When the male genitourinary tract is infected, bacteriotoxin, leukocytes, oxidative stress, and inflammatory factors may change the seminal plasma environment, which leads to the impairment of sperm motility^31^. In 2000, a study showed that the mean reactive oxygen species (ROS) and total antioxidant capacity (ROS-TAC score) of the healthy males was significantly higher than that of patients with chronic prostatitis^32^. Higher ROS and lower TAC induces sperm chromatin structure and sperm plasma membrane damage^33^.

In our study, five articles reported the effect of CBP on sperm motility and four articles reported the percentage of progressively motile sperm respectively^3^,^13^,^14^,^16^,^18^,^19^. These semen parameters were significantly lower than controls (Figure 3 and 4).

A normal semen volume is necessary to carry the sperm into the female reproductive tract, thus semen volume is an important indicator of semen quality. Most of the semen volume is from seminal vesicles, and approximately 1/3 of the volume is contributed by the prostate^34^. However, these different fluids are not mixed until after ejaculation. Hence, the function of prostate and seminal vesicles can effect the semen volume. Additionally, sperm concentration is an important indicator of semen quality, and sperm DNA will be damaged when sperm in a lower sperm density sample^35^. The previous standard considered that a decrease in male fertility was present when the sperm concentration was below the threshold value (15*10^6^/ml to 55*10^6^/ml)^36^, however, according to the WHO manual, a normal sperm concentration was 15*10^6^/ml^25^. Moreover, to some extent, sperm concentration is dependent on semen volume^37^. The results of our study showed that the associations between CBP and semen volume and sperm concentration were not identified (Figure 5,6), different duration of abstinence and smaller samples may explain the reason for these results. Researchers considered that the total sperm count might be a better indicator of normal spermatogenesis^37^, and more studies related to the effect of CBP on the total sperm count are needed.

After ejaculation, semen forms a gel-like coagulum, and during a 10 to 20-min period, the semen liquefies spontaneously^38^. The process is known as coagulation and liquefaction “fibrinolysis”, and can, to a certain extent, be mediated through the high molecular weight seminal vesicle protein system^39^. Many enzymes, which are the essentials of semen liquefaction, and other liquefaction factors are secreted by the spermary and prostate^40^,^41^. Prostate specific antigen (PSA), a prostatic secretion, is responsible for the coagulation and liquefaction of semen when it interacts with high molecular weight seminal vesicle proteins^39^. Hence, prostatitis can change the duration of semen liquefaction. Our study could not identify the association between CBP and the duration of semen liquefaction, because only two studies reported these effects, which limited the statistical power.

Because individual studies had insufficient statistical power, our study involved relatively more cases and controls, enhanced the persuasion and presented a reliable result. What's more, we found a significant negative effect of CBP on basic semen parameters, which may be an available guide for clinical treatment. A drug which can promote sperm vitality, sperm total motility, and percentage of progressively motile sperm may be able to help the men who are acarpous resulting from CBP. However, some limitations of our study should be considered. First, because the included studies were based on case-control design, recall bias and selection bias were inevitable. Second, the number of cases and controls of each study was relatively small, and several studies were excluded due to lack of control data. Third, we could not exclude the possibility that other unmeasured or inadequately measured factors confounded the true effect. Four, our relatively strict inclusion criteria might have introduced selection bias, although little evidence of publication bias was observed.

## Conclusions

Our study suggested a negative effect of CBP on sperm vitality, sperm total motility, and percentage of progressively motile sperm, which may be an available guide for clinical treatment. However, further studies with larger sample sizes on this topic are needed.

## Author Contributions

S.Y.G., L.C.C. and C.D. wrote the main manuscript text; H.G.W. And Y.S.H. prepared all the figures. All authors reviewed the manuscript.

## Figures and Tables

**Table 1:** The characteristics of included studies

Author	Publication year	Country	Mean age(case/controls)	Sample Size(Cases/controls)	Semen parameters
Chen et al.	1998	China	30.53/30.43	17/7	SV, DL,STM,SC
Wang et al.	2000	China	N/N	68/42	SV, SC,STM
Motrich et al.	2005	Argentina	41.41/32.18	15/15	SV, SPV, SPM,STM
Hu et al.	2007	China	38.62/30.45	41/12	SV, SPV, SPM, STM
Hu et al.	2008	China	38.60/32.00	41/12	SV, SPM,STM,SPV
Li et al.	2009	China	N/33	17/30	SV, DL, SPV, SC
Ausmees et al.	2013	Estonia	55.30/56.10	50/35	SV,SC,SPM

**Note**: SV: semen volume; DL: duration of liquefaction; SPM: progressive sperm motility; SPV: sperm vitality; SC: sperm concentration; STM: sperm total motility.
